# Prevalence and determinants of contraceptive method use among Bangladeshi women of reproductive age: a multilevel multinomial analysis

**DOI:** 10.1186/s12889-022-14857-4

**Published:** 2022-12-16

**Authors:** Satyajit Kundu, Subarna Kundu, Md. Ashfikur Rahman, Humayun Kabir, Md. Hasan Al Banna, Saurav Basu, Hasan Mahmud Reza, Ahmed Hossain

**Affiliations:** 1 Global Health Institute, North South University, Dhaka, 1229 Bangladesh; 2 School of Public Health, Southeast University, Nanjing, 210096 China; 3 Faculty of Nutrition and Food Science, Patuakhali Science and Technology University, Patuakhali, 8602 Bangladesh; 4 Statistics Discipline, Khulna University, Khulna, 9208 Bangladesh; 5 Development Studies Discipline, Khulna University, Khulna, 9208 Bangladesh; 6 Department of Public Health, North South University, Dhaka, 1229 Bangladesh; 7 Department of Food Microbiology, Faculty of Nutrition and Food Science, Patuakhali Science and Technology University, Patuakhali, 8602 Bangladesh; 8 Indian Institute of Public Health, New Delhi, 122002 India; 9 Department of Pharmaceutical Sciences, North South University, Dhaka, 1229 Bangladesh; 10 College of Health Sciences, University of Sharjah, 27272 Sharjah, United Arab Emirates

**Keywords:** Prevalence, Contraception use, Multilevel multinomial, Bangladesh, BDHS

## Abstract

**Background:**

Much scholarly debate has centered on Bangladesh's family planning program (FPP) in lowering the country's fertility rate. This study aimed to investigate the prevalence of using modern and traditional contraceptive methods and to determine the factors that explain the contraceptive methods use.

**Methods:**

The study used data from the 2017–18 Bangladesh Demographic and Health Survey (BDHS), which included 11,452 (weighted) women aged 15–49 years in the analysis. Multilevel multinomial logistic regression was used to identify the factors associated with the contraceptive method use.

**Results:**

The prevalence of using modern contraceptive methods was 72.16%, while 14.58% of women used traditional methods in Bangladesh. In comparison to women in the 15–24 years age group, older women (35–49 years) were more unwilling to use modern contraceptive methods (RRR: 0.28, 95% CI: 0.21–0.37). Women who had at least a living child were more likely to use both traditional and modern contraceptive methods (RRR: 4.37, 95% CI: 3.12–6.11). Similarly, given birth in the previous 5 years influenced women 2.41 times more to use modern method compared to those who had not given birth (RRR: 2.41, 95% CI: 1.65–3.52). Husbands'/partners’ decision for using/not using contraception were positively associated with the use of both traditional (RRR: 4.49, 95% CI: 3.04–6.63) and modern methods (RRR: 3.01, 95% CI: 2.15–4.17) rather than using no method. This study suggests rural participants were 21% less likely to utilize modern methods than urban participants (RRR: 0.79, 95% CI: 0.67–0.94).

**Conclusion:**

Bangladesh remains a focus for contraceptive use, as it is one of the most populous countries in South Asia. To lower the fertility rate, policymakers may design interventions to improve awareness especially targeting uneducated, and rural reproductive women in Bangladesh. The study also highlights the importance of male partners’ decision-making regarding women's contraceptive use.

## Introduction

In many countries around the world, sexual and reproductive health (SRH) is a serious public health issue, particularly for women [1]. SRH care is referenced in Goal 3 of the United Nations' Sustainable Development Goals (SDGs), which aims to ensure universal access to sexual and reproductive healthcare services, including family planning, information, and education [1]. Contraception is an unique among medical interventions in terms of the breadth of its good consequences and effectiveness as a method of FP and fertility control, conducive for the betterment of the mother and child the health [2–4]. While family planning (FP) is acknowledged as one of the century's greatest public health successes, and global acceptance is growing [5]. It has been regarded as one of just a few sustainable, cost-effective interventions that can have an instant impact on women and their families and reach far beyond the individual level [6]. As a result of its inclusion in the Millennium Development Goals (MDGs) and Sustainable Development Goals (SDGs) as an indicator for tracking progress on improving maternal health, family planning helps protect women from high-risk pregnancies, unsafe abortions, reproductive tract infections, and sexually transmitted infections (STIs), including HIV/AIDS [7, 8].

At least 200 million women around the world want to utilize family planning that is both safe and effective however, they are unable to do so, resulting in undesired pregnancies [5]. Abortion is performed on more than 50 million of the 190 million women who fall pregnant each year. Increased contraceptive use in poor nations has resulted in a 40% reduction in maternal fatalities over the last two decades just by reducing the number of unintended pregnancies [9]. However, in Bangladesh, the rising trend in contraceptive prevalence rate (CPR) has paused (e.g., 61.0% in 2011, 62.4% in 2014, and 62.0% in 2017), while the lowering trend in total fertility rate (TFR) has also stalled (2.3 children per woman from 2011 to 2017) [10]. The Government of Bangladesh has set a goal of increasing CPR by 75% by 2021, achieving a below replacement level of fertility (i.e., less than 2.1 children per woman), in order to halt population growth and further enhance mother and child health [11]. Despite the declining TFR in Bangladesh has been observed, special attention should be paid to the use of contraceptives by women of reproductive age in this country, as Bangladesh still has a long way to go to reach the target CPR level of more than 70% [12].To sustain the CPR's upward trend by addressing contraceptive use hurdles, the Government of Bangladesh's family planning initiatives must adopt an evidence-based pragmatic approach. Additional efforts are needed to boost CPR and by identifying the factors of using contraceptive methods that have a significant impact on CPR in Bangladesh, this study may contribute to policymaking to reduce the fertility rate [11].

Several previous Bangladeshi studies identified different sociodemographic factors like women’s age, women's educational level, household wealth status, women working status, administrative division, place of residence, religion, number of household members, breastfeeding practice, husband’s education are significantly associated with contraceptive use [4, 13]. While a growing number of literature suggests that parity, autonomy, desire for children, partner communication [14–16], women amenorrheic status, abstaining status, total children born in last five years, and total children ever died have been linked to the use of contraceptives [13]. Preference for sons was also associated with the use of contraceptives among Bangladeshi women [17]. Evidence also shows a significant association between couples’ joint participation in household decision-making and contraceptive use in Bangladesh [18]. A study by Khan et. al., among reproductive aged bangladeshi women reported that having diabetes and hypertension are also linked with the use of contraceptive method, while women having both diabetes and hypertension are more prone to use traditional contraceptive method [19]. Another finding from Bangladesh demonstrates that women's patterns of taking contraception remained unchanged even after experiencing an unexpected pregnancy, while about 54% of women who said they had not taken a contraception before becoming pregnant used modern method after giving birth [20]. Promoting and increasing the contraceptive prevalence rate among women of reproductive age has also been demonstrated to be an effective public health strategy for improving maternal and child health outcomes [21, 22]. Numerous studies demonstrate that increasing CPR directly reduces maternal mortality by reducing unwanted pregnancies, teenage pregnancies, unsafe abortions, and high-risk pregnancies, as well as allowing for pregnancies to be spaced [23, 24].

While several studies have looked at the factors that influence modern contraceptive use only, and very few studies [4, 13, 25] considered both traditional and modern methods; however, these studies applied binary logistic regression after constructing binary outcome variable of current contraception use. Thus, separate determinants of using traditional as well as modern contraception use were not identified among Bangladeshi women.. There is a need to better understand the factors that are associated with the use and choice of method of contraceptives, as the use of contraceptives is suboptimal [26]. Besides, a periodic inspection of prevalence and risk factors is required in order to track its current situation, since high CPR is always expected for controlling births for populous counties like Bangladesh. Therefore, this study would be an addition to fulfill the research gaps. Consequently, this study investigates the prevalence of use of both modern and traditional contraceptive methods and their associated determinants among women of reproductive age in Bangladesh by applying multilevel multinomial logistic regression analysis.

## Methods

### Data source

We used a secondary data set of the most recent Bangladesh Demographic and Health Survey (BDHS) 2017/18 for this study. The survey was carried out from October 2017 to March 2018 under the National Institute of Population Research and Training (NIPORT), and Ministry of Health and Family Welfare, Bangladesh to ascertain the population’s health status. An overview of the ethical procedure, survey procedure, methodology, sampling, and survey tools could be found in the final report of the BDHS 2017/18 which is publicly available [27].

### Sampling and study design

**Fig. 1:**
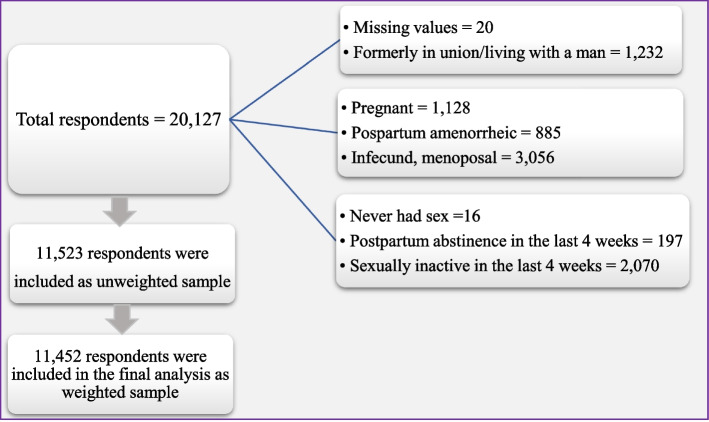
Flow chart of the exclusion criteria and selection of participants (unweighted frequency)

The sampling design of BDHS 2017/18 consisted of a two-stage stratified approach to select the households from a list of enumeration areas (EAs) [27]. Initially, a total of 675 EAs or clusters were selected in this survey as primary sampling units (PSUs). Then, a total of 20,250 households (6,810 in urban and 13,440 in rural) were selected from these PSUs using the stratified sampling method [27]. The married women of reproductive age (15 to 49 years) were interviewed to collect information on the use of contraception methods [27]. From the selected households, 20,376 eligible women were selected for individual interviews from which 20,127 completed their interviews with a response rate of 98.8%. The exclusion criteria of selecting participants have been shown in Fig. 1. Finally, a weighted sample of 11,452 (unweighted 11,523) women who gave information on their contraceptive use was included in the final analysis of this study.

### Outcome measure

Method of contraceptive use among married, non-pregnant Bangladeshi women of reproductive age was the primary outcome variable of this study. The contraceptive methods were categorized into 3 groups: using no method (coded as 0), using the traditional method (coded as 1), and using the modern method (coded as 2). It was assessed based on the participants’ self-report of using contraceptives at the time of the survey by asking the following question: “Are you currently doing something or using any method to delay or avoid getting pregnant?”. Those who responded positively were then asked to indicate the specific method they were using individually or in concurrence with their partner [27]. The methods were considered modern when participants used female sterilization, injections, implant/norplant, pills, intrauterine device (IUD), injection, emergency contraception, female condom, and lactational amenorrhea method (LAM). While the traditional methods included periodic abstinence and withdrawal [27, 28].

### Explanatory variables

The explanatory variables for contraceptive utilization among women were selected on the basis of previous literatures [13, 18, 29, 30] and the availability of the variables in the BDHS 2017–18 dataset. The explanatory variables along with their categories have been shown in Table 1.

**Table 1:** Explanatory variables along with their categories

Sl. no	Variables	Categories
1	Age of women (years)	15–24, 25–34, 35–49
2	Education of women	No education, primary, secondary, higher
3	Women employment status	Working, not working
4	Husband’s education	No education, primary, secondary, higher, don’t know
5	No. of living children	None, 1 – 2, 3 or more
6	Ever had a terminated pregnancy	Yes, no
7	Birth in the last 5 years	No birth, one birth, two or more
8	Decision for using/ not using contraception	Mainly respondents, mainly husband/partner, joint decision
9	Desire for more children	Want more, don’t want more
10	Knowledge of ovulatory cycle	Yes, no
11	Household wealth status	Poor, Middle, Rich
12	Place of residence	Rural, Urban
13	Administrative division	Barishal, Chittagong, Dhaka, Khulna, Mymensingh, Rajshahi, Rangpur, Sylhet

### Statistical analysis

Both unweighted and weighted frequencies and percentages were calculated to show the background characteristics of study participants. Considering the complex survey of BDHS, we used the *“svy”* command in STATA version 17.0 (StataCorp, College Station, TX, USA) for assigning the sample weight to adjust for clustering effect and sample stratification. Since the BDHS 2017/18 used a two-stage stratified cluster sampling having a hierarchical composition, a multilevel regression model would be appropriate to consider the cluster variation in the analysis [31]. Thus, to consider the cluster effect in the analysis, the multilevel multinomial regression model was used to identify the association between outcome and explanatory variables where clusters (EAs) were considered as level-2 factors. We used generalized structural equation modeling (GSEM) using the “*gsem”* command in STATA to estimate the model. The GSEM model assessed the fixed effects of various explanatory variables and also assessed random effects at the cluster level. Multicollinearity among explanatory variables was checked using variance inflation factor (VIF). The adjusted relative risk ratio (RRR) along with 95% confidence interval (CI) were interpreted and statistical significance was considered when a p-value was less than 0.05.

## Results

**Fig. 2:**
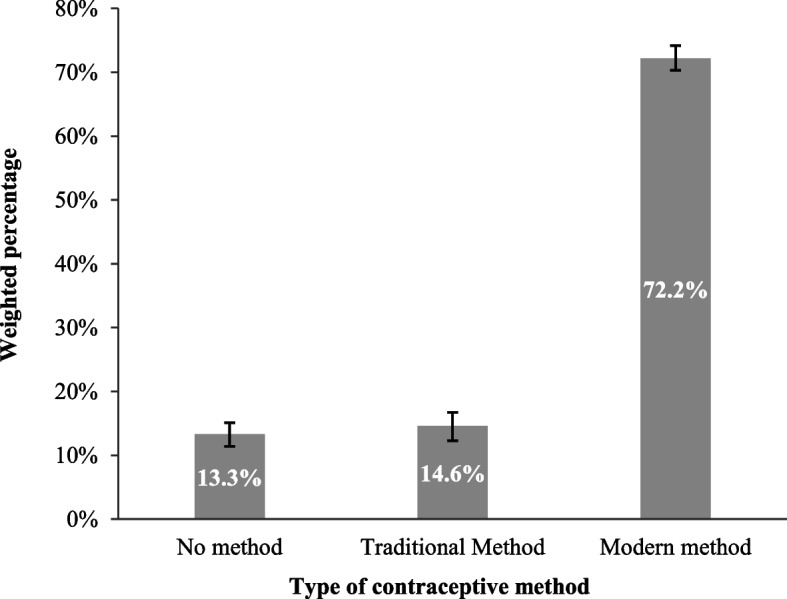
Prevalence of type of contraceptive method used by Bangladeshi women

Background characteristics of study participants are presented in Table 2. A total of 11,452 (weighted) participants were included in the present study. The majority of the women (38.8%) were aged between 25 and 34 years, and 41.2% completed secondary education. Half of them (50.5%) were employed, and 56.2% had 1 to 2 living children. More than half of them (57.7%) had no birth in 5 years. More than two-thirds of them (78.4%) use contraception based on the joint decision. The women do not want to take more children was 65.0%. The maximum of them (42.2%) was rich in wealth household index, and almost one-third (69.4%) of them was from rural areas. Women from the Dhaka division were the highest (26.6%) participants. We found that the prevalence of using modern, and traditional contraceptive methods among Bangladeshi women were 72.2% (95% CI: 70.3–74 0.2) and 14.6% (95% CI: 12.3–16.7), respectively. While about 13.0% of participants did not use any contraceptive method (Fig. 2).

**Table 2:** Background characteristics of study participants

Variables	Unweighted		Weighted	
	**Frequency**	% (SE)	**Frequency**	% (SE)
**Overall**	11,523		11,452	
**Age of women**				
15–24 years	3195	27.7 (0.4)	3244	28.3 (0.4)
25–34 years	4492	39.0 (0.5)	4445	38.8 (0.5)
35–49 years	3836	33.3 (0.4)	3763	32.9 (0.4)
**Education of women**				
No education	1463	12.7 (0.3)	1542	13.5 (0.3)
Primary	3581	31.1 (0.4)	3580	31.3 (0.4)
Secondary	4664	40.5 (0.5)	4722	41.2 (0.4)
Higher	1815	15.8 (0.3)	1607	14.0 (0.3)
**Women employment status**				
Working	5778	50.1 (0.5)	5784	50.5 (0.5)
Not working	5745	49.9 (0.5)	5668	49.5 (0.5)
**Husbands’ education**				
No education	2322	20.2 (0.3)	2356	20.6 (0.3)
Primary	3631	31.5 (0.4)	3714	32.4 (0.4)
Secondary	3325	28.9 (0.4)	3344	29.2 (0.4)
Higher	2227	19.3 (0.3)	2015	17.6 (0.3)
Don’t know	18	0.2 (0.0)	23	0.2 (0.0)
**No. of living children**				
None	970	8.4 (0.2)	985	8.6 (0.2)
1 – 2	6538	56.7 (0.5)	6436	56.2 (0.5)
3 or more	4015	34.8 (0.4)	4031	35.2 (0.4)
**Ever had a terminated pregnancy**				
Yes	2453	21.3 (0.3)	2354	20.6 (0.4)
No	9070	78.7 (0.4)	9098	79.4 (0.4)
**Birth in the last 5 years**				
No birth	6659	57.8 (0.5)	6611	57.7 (0.5)
One birth	4231	36.7 (0.4)	4226	36.9 (0.4)
Two or more	633	5.5 (0.2)	614	5.4 (0.2)
**Decision for using/not using contraception**				
Mainly respondents	1730	15.0 (0.3)	1736	15.2 (0.3)
Mainly husband/partner	743	6.5 (0.2)	742	6.5 (0.2)
Joint decision	9050	78.5 (0.4)	8974	78.4 (0.4)
**Desire for more children**				
Want more	4000	34.7 (0.4)	4007	35.0 (0.4)
Don’t want more	7523	65.3 (0.4)	7445	65.0 (0.5)
**Knowledge of ovulatory cycle**				
Yes	4245	36.8 (0.4)	4140	36.2 (0.4)
No	7278	63.2 (0.5)	7312	63.9 (0.5)
**Household wealth status**				
Poor	4403	38.2 (0.4)	4408	38.5 (0.4)
Middle	2135	18.5 (0.3)	2209	19.3 (0.3)
Rich	4985	43.3 (0.5)	4834	42.2 (0.4)
**Place of residence**				
Rural	7057	61.2 (0.4)	7942	69.4 (0.4)
Urban	4466	38.8 (0.4)	3510	30.7 (0.4)
**Administrative division**				
Barisal	1156	10.0 (0.3)	595	5.2 (0.2)
Chittagong	1457	12.6 (0.3)	1783	15.6 (0.3)
Dhaka	1800	15.6 (0.3)	3044	26.6 (0.4)
Khulna	1528	13.3 (0.3)	1332	11.6 (0.3)
Mymensingh	1367	11.9 (0.3)	973	8.5 (0.2)
Rajshahi	1563	13.6 (0.3)	1677	14.6 (0.3)
Rangpur	1522	13.2 (0.3)	1450	12.7 (0.3)
Sylhet	1130	9.8 (0.2)	597	5.2 (0.2)

*SE* Standard Error

The variations in using contraception methods among Bangladeshi women by the selected individual, household, and community variables are presented in Table 3. Looking at the bivariate association, all the explanatory variables except the place of residence were significantly associated with the use of contraceptive methods of women.

**Table 3:** Variations in patterns of using contraception methods among Bangladeshi women by selected explanatory variables

Variables	Contraceptive method (weighted %, row)			*P* value
	**No method**	**Traditional method**	**Modern method**	
**Overall prevalence (95% CI)**	13.3 (11.4–15.1)	14.6 (12.3–16.7)	72.2 (70.3–74.2)	
**Age of women**				
15–24 years	21.9	7.7	70.4	< 0.001
25–34 years	12.4	10.1	77.5	
35–49 years	6.9	25.8	67.3	
**Education of women**				
No education	6.9	23.1	70.0	< 0.001
Primary	11.3	15.9	72.9	
Secondary	15.2	10.9	74.0	
Higher	18.2	14.4	67.4	
**Women employment status**				
Yes	14.9	14.0	71.1	< 0.001
No	11.6	15.2	73.2	
**Husbands’ education**				
No education	8.9	17.9	73.2	< 0.001
Primary	13.0	13.0	74.0	
Secondary	15.4	12.5	72.1	
Higher	14.9	17.1	68.0	
Don’t know	44.2	15.0	40.8	
**No. of living children**				
None	52.4	8.1	39.4	< 0.001
1–2	12.4	12.1	75.4	
3 or more	5.0	20.0	74.9	
**Ever had a terminated pregnancy**				
Yes	14.2	18.2	67.6	< 0.001
No	13.0	13.6	73.3	
**Birth in the last 5 years**				
No birth	16.4	18.7	64.9	< 0.001
One birth	9.3	9.1	81.6	
Two or more	6.5	8.0	85.5	
**Decision for using/not using contraception**				
Mainly respondents	13.9	12.8	73.3	0.007
Mainly husband/partner	10.4	17.1	72.5	
Joint decision	13.4	14.7	71.9	
**Desire for more children**				
Want more	31.0	8.1	60.9	< 0.001
Don’t want more	3.7	18.0	78.2	
**Knowledge of ovulatory cycle**				
Yes	11.1	19.0	69.9	< 0.001
No	14.5	12.1	73.4	
**Household wealth status**				
Poor	12.5	14.3	73.3	0.048
Middle	13.8	14.0	72.2	
Rich	13.8	15.1	71.1	
**Place of residence**				
Rural	13.5	14.7	71.8	0.256
Urban	12.7	14.3	72.9	
**Administrative division**				
Barisal	11.7	15.9	72.4	< 0.001
Chittagong	12.9	15.1	72.1	
Dhaka	13.5	13.7	72.8	
Khulna	11.9	18.5	69.6	
Mymensingh	15.2	11.6	73.2	
Rajshahi	14.8	12.9	72.3	
Rangpur	12.1	14.6	73.4	
Sylhet	13.2	17.0	69.8	

*CI* Confidence Interval

In Table 4, results from the multilevel multinomial regression model show the likelihood (presented as relative risk ratio [RRR]) of using traditional, and modern contraceptive methods relative to using no contraceptive method. Table 4 also presents the random effects (measured as variance) at the cluster level. Compared to the younger aged women (15–24 years), the older aged women, 25–34 years, had significantly 49% (RRR: 0.51, CI: 0.39, 0.66) lower possibilities of using the traditional contraceptive method, while women aged 25–34 years and 35–49 years were 53% (RRR: 0.47, CI: 0.39, 0.56) and 72% (RRR: 0.28, CI: 0.21, 0.37) less likely to use the modern contraceptive method, respectively. Women with secondary education were 30% (RRR: 0.70, CI: 0.51, 0.97) less likely to use the traditional method compared to those having no education. However, the likelihood of traditional method use was 45% (RRR: 1.45, CI: 1.02, 2.05) higher among those whose husbands' education was higher compared to illiterate husbands.

**Table 4:** Results of the multilevel multinomial regression of contraceptive use on selected explanatory variables among Bangladeshi women

Variables	Using traditional method vs. using no method		Using modern method vs. using no method	
	**RRR**	**95% CI**	**RRR**	**95% CI**
**Fixed-effect results**				
**Age of participants** (RC = 15–24 years)				
25–34 years	0.51^***^	0.39, 0.66	0.47^***^	0.39, 0.56
35–49 years	0.76	0.54, 1.07	0.28^***^	0.21, 0.37
**Education** (RC** = **No education)				
Primary	0.77	0.57, 1.03	0.97	0.74, 1.27
Secondary	0.70^*^	0.51, 0.97	1.09	0.82, 1.45
Higher	0.79	0.52, 1.19	1.25	0.89, 1.78
**Employment status** (RC = Not working)				
Working	0.96	0.81, 1.14	1.08	0.93, 1.24
**Husbands’ education** (RC = No education)				
Primary	0.88	0.69, 1.13	0.89	0.72, 1.10
Secondary	0.88	0.67, 1.16	0.84	0.66, 1.06
Higher	1.45^*^	1.02, 2.05	0.92	0.69, 1.23
Don’t know	0.48	0.10, 2.40	0.18^*^	0.05, 0.68
**No. of living children** (RC = None)				
1–2	3.13^***^	2.21, 4.45	3.54^***^	2.81, 4.47
3 or more	3.96^***^	2.54, 6.17	4.37^***^	3.12, 6.11
**Ever had a terminated pregnancy** (RC = No)				
Yes	0.77^**^	0.63, 0.93	0.64^***^	0.55, 0.75
**Birth in the last 5 years** (RC = No birth)				
One birth	1.52^***^	1.21, 1.90	2.49^***^	2.07, 2.98
Two or more	1.34	0.83, 2.15	2.41^***^	1.65, 3.52
**Decision for using/ not using contraception** (RC = Mainly respondents)				
Mainly husband/partner	4.49^***^	3.04, 6.63	3.01^***^	2.15, 4.17
Joint decision	2.34^***^	1.86, 2.94	1.78^***^	1.48, 2.15
**Desire for more children** (RC = Want more)				
Don’t want more	18.10^***^	13.87, 23.61	20.16^***^	16.19, 25.12
**Knowledge of ovulatory cycle** (RC = No)				
Yes	1.93^***^	1.63, 2.29	1.19^*^	1.04, 1.38
**Household wealth status** (RC = Poor)				
Middle	0.93	0.73, 1.17	0.99	0.82, 1.20
Rich	1.11	0.88, 1.41	1.12	0.92, 1.36
**Place of residence** (RC = Urban)				
Rural	0.86	0.71, 1.04	0.79^**^	0.67, 0.94
**Administrative division** (RC = Barisal)				
Chittagong	0.69^*^	0.48, 0.99	0.74	0.54, 1.02
Dhaka	0.70	0.49, 1.00	0.85	0.62, 1.15
Khulna	1.10	0.77, 1.57	0.93	0.68, 1.28
Mymensingh	0.51^***^	0.35, 0.73	0.78	0.57, 1.07
Rajshahi	0.60^**^	0.42, 0.85	0.77	0.57, 1.05
Rangpur	0.78	0.55, 1.12	0.82	0.60, 1.13
Sylhet	0.78	0.53, 1.14	0.68^*^	0.48, 0.95
**Random-effect results**				
Cluster random effects (95% CI) ^a^	0.133^***^	0.049, 0.361	0.191^***^	0.112, 0.327

*RRR* Relative Risk Ratio, *CI* Confidence Interval, *RC* Reference Category

^*^ *P* < 0.05

^**^ *P* < 0.01

^***^ *P* < 0.001

^a^ Significance of cluster-level random effects was assessed using log-likelihood ratio tests (comparing models with and without random effects)

Women who had at least a living child were 3.13 (RRR: 3.13, CI: 2.21, 4.45) times more likely to use the traditional method compared to the women who had no living child. Moreover, the likelihood of using the modern method was 3.54 (RRR: 3.54, CI: 2.81, 4.47) times higher among the women who had at least a living child compared to those who had no living child. Women who ever experienced terminated pregnancy were 23% (RRR: 0.77, CI: 0.63, 0.93) and 36% (RRR: 0.64, CI: 0.55, 0.75) less likely to use the traditional and modern method, respectively, compared to those who didn’t experience terminated pregnancy ever. Women giving one birth in the last 5 years were 1.52 (RRR: 1.52, CI: 1.21, 1.90) times more likely to use the traditional method compared to those who had no birth. Similarly, women who gave one birth, and two or more birth were 2.49 (RRR: 2.49, CI: 2.07, 2.98) and 2.41 (RRR: 2.41, CI: 1.56, 3.52) times more prone to use the modern method (Table 4).

The likelihood of using traditional and modern methods were 4.49 (RRR: 4.49, CI: 3.04, 6.63) times and 3.01 (RRR: 3.01, CI: 2.15, 4.17) times higher, respectively, while the decision to use contraception depended on husband/partner compared to the decision taken by women mainly. When the decision was taken jointly, the likelihood of using the traditional and modern method was 2.34 (RRR: 2.34, CI: 1.86, 2.94) times and 1.78 (RRR: 1.78, CI: 1.48, 2.15) times higher, respectively. The women who did not want to take more children were 18.10 (RRR: 18.10, CI: 13.87, 23.61) times and 20.16 (RRR: 20.16, CI: 16.19, 25.12) times more likely to use the traditional and modern method, respectively than those with a desire for more children. Women knowing the ovulatory cycle were 1.93 (RRR: 1.93, CI: 1.63, 2.29) times and 1.19 (RRR: 1.19, CI: 1.04, 1.38) times more likely to use the traditional and modern method, respectively, than those who had not. The rural women were 21% (RRR: 0.79, CI: 0.67, 0.94) less likely to use the modern method than women from urban areas. Compared to the women from the Barisal division, the likelihood of using the modern method was 72% (RRR: 0.68, CI: 0.48, 0.95) less among the women from the Sylhet division. Finally, the cluster random effect was significant for traditional contraceptive method use versus using no method, and for modern contraceptive method versus using no method (Table 4).

## Discussion

The study assessed the method of contraceptive use and their predictors among married, non-pregnant Bangladeshi women of the reproductive (15–49 years) age group using the current Bangladesh demographic and health survey dataset (2017–18). In this study, we found 72% of participants were using modern contraceptive method, indicating an increase from 62% found in 2014 among Bangladeshi women [13]. Similar to the modern method, use of traditional contraceptive method is also increased to 14.6% in this study from 8.1% found in 2014 [13], while about 37% Bangladeshi women didn’t use any contraceptive method in 2014 [13], and this figure has reduced to 13% in the present study. Since Bangladesh aimed to have a 75% contraceptive prevalence by 2020, the Health, Population and Nutrition Sector Development Program (HPNSDP) of Bangladesh established strategic goals to enhance the general use of family planning by making family planning services available, acceptable, and cheap to all men and women of reproductive age [32, 33]. These could be plausible reasons of increasing use of contraceptive method in Bangladesh.

The use of modern contraception methods was reported by more than two in three women with the highest use observed in the 25–34 age group. According to a previous study [34], young girls and women are more likely to plan to take contraception which corroborates the findings of the present study. The fact that young women may not intent to be pregnant early, while many older women are not sexually active or have reduced their coital frequency may be connected to older women's decreased propensity to utilize contraception [34]. Sexually active female adolescents from other LMICs in Asia and Africa also frequently experience societal and health system barriers in accessing contraception that is linked to unwanted pregnancies and adverse pregnancy outcomes [35, 36].

In this study, education status of the women or even their husbands were not independently associated with the decision for acceptance of modern contraception methods a finding similar to that observed in the previous BDHS (2011–14) [13]. However, globally, several studies amongst LMICs in Asia and Africa suggest increased demand for modern contraceptive methods with higher educational status of women that often correlates with their desire for career planning and development [30, 37, 38]. Moreover, a previous study from Nepal [39] suggests that substantially higher educational status of husbands compared to wives is an independent predictor for their acceptance of condoms for routine contraception purposes. A previous round of the BDHS (2011) had found that contraception use was higher in the employed women in contradiction to the current round where contraception use was greater in the unemployed women [25]. Furthermore, the positive employment status of the married women in the present study did not indicate any statistically significant increase in their use of any contraception method after adjustment for covariates.

Women in this study having 3 or more children were more likely to use both MC and traditional methods compared to those having no living children, a factor that strongly correlates with the desire to have no more children, indicative of the achievement of desired family size. A previous study in East African countries also argued that women who have more children are more likely to use a modern contraceptive method [38]. Previous study also suggests that completion of desired family size is a reliable indicator of willingness to use MC methods [38]. However, one in five women in this group were still using only traditional methods of contraception which have a high risk of failure and unintended pregnancies. Consequently, family planning programs in the country may encourage women using traditional conraceptive methods to switch to those modern methods that are compatible with their socio-cultural milieu [40]. In the current study, women's preference for having children was a significant factor of contraceptive usage. Contraception use was more prevalent among women who didn't desire more children than among those who did. This result is consistent with the earlier research [4, 41].

In this study, women with a history of terminated pregnancy were significantly less likely to use any contraceptive method. This finding is in line with the previous study in Nepal [42], and Malawi [34]. However, the inclusion of past experiences with terminated pregnancies as a factor warrants further discussion because it might either be an unwanted naturally terminated pregnancy (miscarriage) or one that was purposefully terminated [34].

In this study, the use of both modern and traditional methods was reported by the women as primarily a decision by the husband and significantly less likely to be a joint decision. However, similar to the BDHS 2011 round, the practice of spousal joint decision making was observed to increase the likelihood of practicing modern contraceptive methods [18].However, a contradictory finding was observed in another study where women who were empowered to exercise sexual autonomy were most likely to enhance their capacity to utilize modern contraception regardless of their educational levels [43]. This finding demonstrate the necessity of involvement of men in the Bangladesh family planning and reproductive health programmes has been long recognized for nearly three decades and the present analysis also indicates that men are the primary decision-makers in the Bangladeshi society with regards to determining their family size and the acceptance of contraception by their wives [13, 44].

Awareness of the ovulation period was a significant predictor of any type of contraception use by women in this study, a finding corroborated by evidence from previous studies where the authors identified that women who knew their ovulation cycle were more likely to use contraception than those who did not know their cycle [45, 46]. The likelihood of using contraception to prevent conception during the ovulatory phase may be higher among women who are aware of their ovulatory cycle than among those who are unaware of it [46].. Future research needs to be directed towards understanding the association between choice of contraception method and knowledge of ovulatory cycle so that the policymakers could assess whether interventions designed to improve the knowledge of the reproductive cycle in Bangladeshi women could enhance their acceptability of modern contraceptive methods.

According to the study, women in rural regions use modern contraception less frequently than those in urban. This result was in line with earlier research conducted in Bangladesh [4] and Ethiopia [29, 47]. This can be due to rural women's inferior access to maternal health care service, such as contraception [29]. Another possible causes for observed inequalities in contraceptive usage between rural and urban areas include differences in cultural views and beliefs [46]. Besides, Urban women are more likely to use any type of contraception and have better access to contraception as a whole [48].

One of the strengths of this study was using the most recent nationally representative (BDHS 2017–18) data set. And also, this study used the multilevel model to consider the cluster effect on the determinant factors of contracetion methods use in Bangladesh which is the appropriate analysis approach for such data set. We identified the determinats of both traditional and modern contraception methods among Bangladeshi women. There are a few study limitations. First, data on some variables such as joint families and the opinion of mothers-in-law regarding contraception use were not captured. Similarly, the opinion of men regarding their acceptance of barrier methods of contraception by themselves or any modern contraception methods by their female partners was also not included.

## Conclusions

The study finds about one-fourth of Bangladeshi women of reproductive age did not use any form of modern contraception. Women with 3 or more children and those having awareness of women’s ovulatory cycle were significantly more likely to use contraceptive methods, However, women's education, occupation, and household wealth did not significantly improve their likelihood of using any contraception method. Husbands were also the key drivers of decision-making in regulating the fertility of their wives but shared decision-making by both husband and wife considerably augmented the acceptability of modern contraception technologies. These findings suggest that women's empowerment for health promotion requires delicate messaging that encourages their participation in planning and deciding on their family size while reducing taboos and misconceptions about modern contraception methods.

## Data Availability

The data that support the findings of this study are available from Demographic and Health Surveys (DHS) Program dataset of Bangladesh (https://dhsprogram.com/) but restrictions apply to the availability of these data, which were used under license for the current study, and so are not publicly available. Data are however available from the authors upon reasonable request and with permission of Bangladesh Demographic and Health Surveys (BDHS) Program.

## Abbreviations

SRH: Sexual and Reproductive Health
SDG: Sustainable Development Goal
FP: Family Planning
STI: Sexually Transmitted Infections
CPR: Contraceptive Prevalence Rate
TFR: Total Fertility Rate
BDHS: Bangladesh Demographic and Health Survey
EAs: Enumeration Areas
PSU: Primary Sampling Unit
RRR: Relative Risk ratio
CI: Confidence Interval
WHO: World Health Organization
